# Serum neurofilament light protein correlates with unfavorable clinical outcomes in hospitalized patients with COVID-19

**DOI:** 10.1126/scitranslmed.abi7643

**Published:** 2021-07-14

**Authors:** Mercedes Prudencio, Young Erben, Christopher P. Marquez, Karen R. Jansen-West, Camila Franco-Mesa, Michael G. Heckman, Launia J. White, Judith A. Dunmore, Casey N. Cook, Meredith T. Lilley, Yuping Song, Caroline F. Harlow, Björn Oskarsson, Katharine A. Nicholson, Zbigniew K. Wszolek, LaTonya J. Hickson, John C. O’Horo, Jonathan B. Hoyne, Tania F. Gendron, James F. Meschia, Leonard Petrucelli

**Affiliations:** 1 Department of Neuroscience, Mayo Clinic, Jacksonville, FL 32224, USA.; 2 Neuroscience Graduate Program, Mayo Clinic Graduate School of Biomedical Sciences, Jacksonville, FL 32224, USA.; 3 Division of Vascular and Endovascular Surgery, Mayo Clinic, Jacksonville, FL 32224, USA.; 4 Department of Laboratory Medicine and Pathology, Mayo Clinic, Jacksonville, FL 32224, USA.; 5 Division of Biomedical Statistics and Informatics, Mayo Clinic, Jacksonville, FL 32224, USA.; 6 Department of Neurology, Mayo Clinic, Jacksonville, FL 32224, USA.; 7 Sean M. Healey and AMG Center for ALS, Massachusetts General Hospital (MGH), Boston, MA 02114, USA.; 8 Division of Nephrology and Hypertension, Mayo Clinic, Jacksonville, FL 32224, USA.; 9 Division of Infectious Diseases, Mayo Clinic, Rochester, MN 55905, USA.; 10 Division of Pulmonary and Critical Care Medicine, Mayo Clinic, Rochester, MN 55905, USA.

## Abstract

Brain imaging studies of patients with COVID-19 show evidence of macro- and microhemorrhagic lesions, multifocal white matter hyperintensities, and lesions consistent with posterior reversible leukoencephalopathy. Imaging studies, however, are subject to selection bias, and prospective studies are challenging to scale. Here, we evaluated whether serum neurofilament light chain (NFL), a neuroaxonal injury marker, could predict the extent of neuronal damage in a cohort of 142 hospitalized patients with COVID-19. NFL was elevated in the serum of patients with COVID-19 compared to healthy controls, including those without overt neurological manifestations. Higher NFL serum concentrations were associated with worse clinical outcomes. In 100 hospitalized patients with COVID-19 treated with remdesivir, a trend toward lower NFL serum concentrations was observed. These data suggest that patients with COVID-19 may experience neuroaxonal injury and may be at risk for long-term neurological sequelae. Neuroaxonal injury should be considered as an outcome in acute pharmacotherapeutic trials for COVID-19.

## Brain damage marker in COVID-19

SARS-CoV-2 infection, the cause of coronavirus disease 2019 (COVID-19), causes neurological manifestations in a substantial proportion of patients. Determining the extent of neuronal injury is essential to better understand disease pathophysiology and to evaluate potential therapies. Prudencio *et al.* analyzed serum from 142 patients hospitalized with COVID-19 and showed that the expression of the neurofilament light protein (NFL), a marker of neuroaxonal injury, was elevated compared to healthy controls. In addition, serum NFL expression correlated with disease severity and tended to be reduced in subjects treated with remdesivir. The results suggest that serum NFL analysis should be incorporated when evaluating therapeutic trials for COVID-19.

## Teaser

Hospitalized patients with COVID-19 show increased serum concentrations of neurofilament light chain that correlate with worse clinical outcomes.

## INTRODUCTION

Since the World Health Organization declared a global pandemic on 11 March 2020, severe acute respiratory syndrome coronavirus 2 (SARS-CoV-2) has caused more than 2.8 million deaths worldwide and more than 550,000 deaths within the United States (*1*). Although common symptoms of coronavirus disease 2019 (COVID-19) include fever, cough, fatigue, and shortness of breath, the manifestations of COVID-19 can vary widely. For example, some patients can develop pneumonia, acute respiratory distress syndrome, myocardial injury, arrhythmias, and/or multiorgan failure (*2*), and it is increasingly recognized that SARS-CoV-2 can cause neurologic signs (*3*–*10*). Clinical studies observed neuropsychiatric manifestations in up to 70% of patients with COVID-19, including young adults and patients in whom respiratory symptoms have long resolved (*10*–*12*). Brain imaging studies detected diverse lesions including perfusion abnormalities with or without acute infarctions, cerebral venous thrombosis, macro- and microhemorrhages, multifocal white matter and basal ganglia lesions, meningeal enhancement, central pontine myelinolysis, posterior reversible encephalopathy syndrome, and neuritis (*13*–*16*). Moreover, brain autopsy studies found SARS-CoV-2 RNA or proteins in various neuroanatomical regions of patients with COVID-19, with astrogliosis, activation of microglia, and infiltration of cytotoxic T lymphocytes noted in many cases (*17*, *18*).

Given that neurologic manifestations are now considered common features of COVID-19, we sought to examine the utility of serum neurofilament light chain (NFL) in assessing the frequency, severity, and clinical consequences of neuronal injury associated with SARS-CoV-2 infections warranting hospitalization. Recently, we determined that blood NFL concentrations are associated with radiographic markers of brain tissue damage, as well as indicators of neurological, functional, and cognitive status in patients with ischemic or hemorrhagic stroke (*19*). On the basis of these findings and others (*20*) establishing NFL as a marker of axonal injury, we investigated whether serum NFL was elevated in hospitalized patients with COVID-19 and whether it correlated with clinical outcomes and disease severity.

## RESULTS

### Serum NFL is elevated in hospitalized patients with COVID-19

**Fig. 1:**
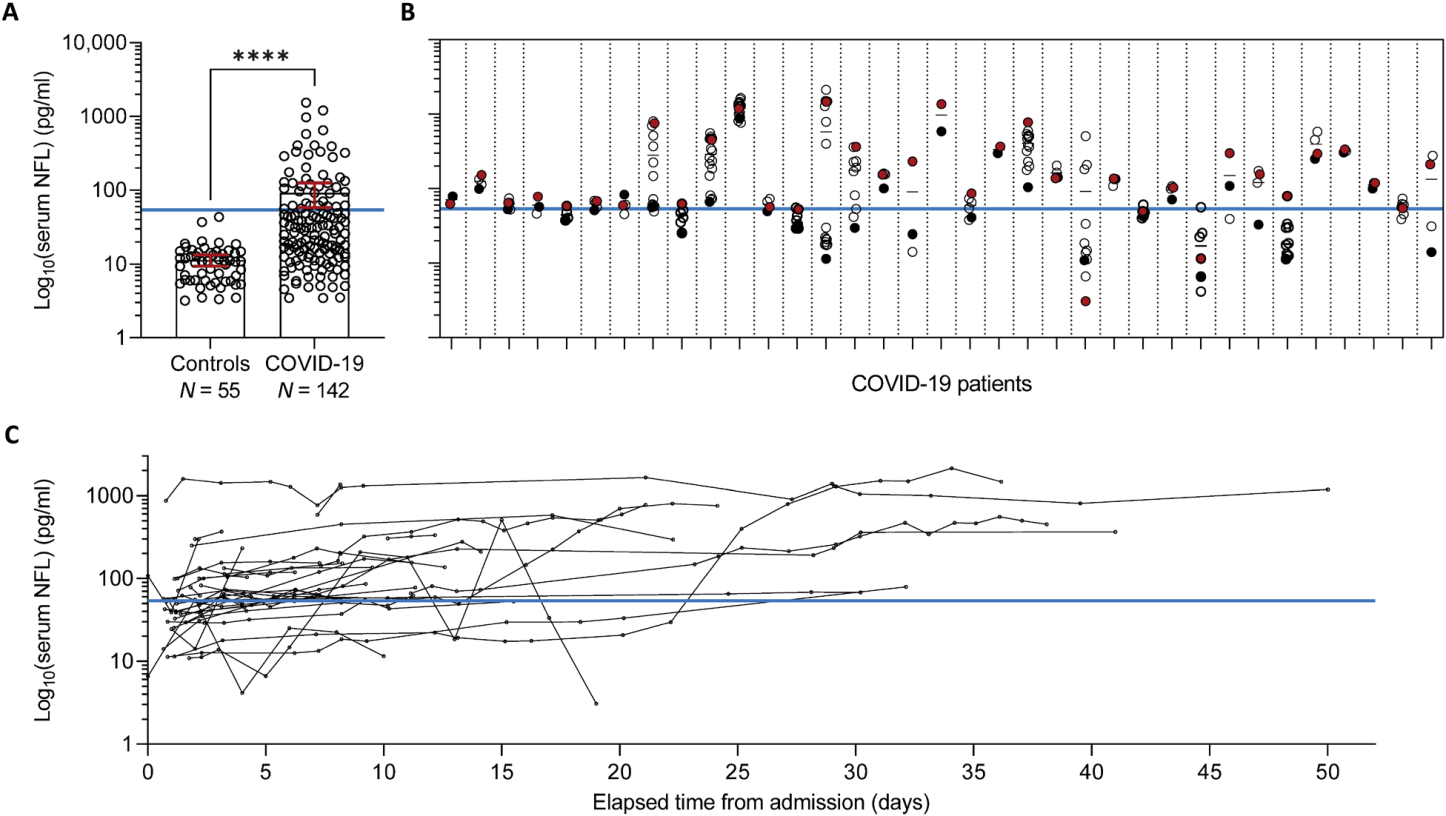
Serum NFL is elevated in hospitalized patients with COVID-19. (**A**) Comparison of serum NFL between healthy controls (Controls, *N* = 55) and hospitalized patients with COVID-19 (mean NFL per patient, *N* = 142). Bars represent mean NFL concentrations with 95% CIs. Statistical differences were assessed using a stratified van Elteren Wilcoxon rank sum test, where the test was stratified by both age as a four-level categorical variable (based on sample quartiles) and sex. ****P < 0.001. (**B**) Serum NFL in 35 patients to highlight the range of NFL concentrations in longitudinally collected samples. The first and last NFL concentrations are indicated by a black and red dot, respectively, and mean concentrations for each patient are presented by the small black solid horizontal lines. (**C**) Serum NFL trends over time in the 35 patients with COVID-19 with longitudinal blood draws and for whom at least one NFL measurement is at least 3 SDs above the group mean of NFL in control individuals. NFL concentrations are shown on the base 10 logarithmic scale. The NFL concentration representing the mean control NFL + 3 SDs is indicated by a solid blue horizontal line.

We measured NFL in 488 serum samples longitudinally collected from 142 hospitalized patients with COVID-19 and in cross-sectional serum samples from 55 healthy controls. A summary of patient demographics, treatments, and clinical outcomes is shown in Table 1. Characteristics of control individuals are presented in table S1. The median age in patients with COVID-19 was 62 years (range, 22 to 99 years), and 57.7% were male (82 patients). In control individuals, the median age was 62 years (range, 32 to 84 years) and 50.9% were male (28 patients). Comparing mean NFL concentrations for each of the 142 patients with COVID-19 to NFL concentrations of the 55 controls revealed that serum NFL was significantly higher in patients (median, 29.4 pg/ml; range, 3.4 to 1538.4 pg/ml) than controls (median, 10.9 pg/ml; range, 3.2 to 43.2 pg/ml; *P* < 0.001; Fig. 1A). This finding was consistent after correcting for age and sex and when examining the first, last, minimum, or maximum NFL measurement per patient (Table 2). To assess how many patients with COVID-19 had elevated NFL according to a prespecified cutoff, we determined the number of patients with an NFL concentration of at least 3 SDs above the group mean NFL concentration of control individuals. For this analysis, we focused on maximum NFL measurements given that NFL concentrations fluctuate between time points, as demonstrated for 35 of the patients with longitudinal NFL data (Fig. 1B), such that using mean NFL concentrations could mask our ability to discern elevated NFL concentrations at one or more time points. About 34% of patients with COVID-19 (48 individuals) had NFL concentrations higher than the cutoff. We additionally determined the number of patients with an NFL concentration of at least 2 SDs above the mean control concentration and found that 53% of patients (76 individuals) met this criterion.

**Table 1:** Characteristics, treatment, and outcomes for patients with COVID-19. ICU, intensive care unit; BMI, body mass index; CKD, chronic kidney disease; LOS, length of hospital stay; mRS, modified Rankin scale.

**Variable**	***N***	**Median (minimum, maximum) or no. (%)**
Patient characteristics		
Age at admission (years)	142	62 (22, 99)
Sex (male)	142	82 (57.7%)
Race	142	
White		96 (67.6%)
Black		32 (22.5%)
Asian		12 (8.5%)
Other or not disclosed		2 (1.4%)
BMI	142	30.0 (15.3, 80.1)
Obesity	142	71 (50.0%)
CKD	142	17 (12.0%)
Length of time from admission to first NFL (days)	142	1 (0, 32)
Number of NFL measurements	142	
1		42 (29.6%)
2		20 (14.1%)
3		29 (20.4%)
4		24 (16.9%)
5		8 (5.6%)
>5		19 (13.4%)
Treatments		
Monoclonal antibody	142	37 (26.1%)
Remdesivir	142	106 (74.6%)
Dexamethasone therapy	142	116 (81.7%)
Convalescent plasma	142	44 (31.0%)
Outcomes		
Mechanical ventilation (intubation)	142	30 (21.1%)
ICU admission	142	54 (38.0%)
LOS (days)	142	9 (2, 119)
Death	142	13 (9.2%)
mRS at discharge	142	
0		1 (0.7%)
1		40 (28.2%)
2		35 (24.6%)
3		33 (23.2%)
4		15 (10.6%)
5		7 (4.9%)
6		11 (7.7%)

**Table 2:** Comparison of serum NFL between healthy controls and patients with COVID-19. In unadjusted analyses, *P* values result from a Wilcoxon rank sum test. In adjusted analyses, *P* values result from a stratified van Elteren Wilcoxon rank sum test, where the test was stratified by both age as a four-level categorical variable (based on sample quartiles) and sex. N/A, not applicable.

	***N***	**Median (minimum,** **maximum) NFL** **concentration**	***P* value vs. controls**	
			**Unadjusted**	**Adjusting for age and sex**
Healthy controls	55	10.9 (3.2, 43.2)	N/A	N/A
Patients with COVID-19				
First NFL measurement	142	21.8 (2.9, 1538.4)	<0.001	<0.001
Final NFL measurement	142	32.3 (2.2, 1538.4)	<0.001	<0.001
Mean NFL per patient	142	29.4 (3.4, 1538.4)	<0.001	<0.001
Minimum NFL per patient	142	20.3 (2.2, 1538.4)	<0.001	<0.001
Maximum NFL per patient	142	37.9 (3.4, 2131.0)	<0.001	<0.001

### Elevated serum NFL associates with worse clinical outcomes in hospitalized patients with COVID-19

We next examined the relationship between serum NFL concentrations and diverse clinical outcomes: mechanical ventilation (intubation), admission to the intensive care unit (ICU), length of hospital stay (LOS), and modified Rankin scale (mRS) at discharge. As shown in Table 3, in unadjusted analyses and in analyses adjusting for age, sex, body mass index (BMI), and chronic kidney disease (CKD), serum NFL concentrations were significantly higher in patients who needed mechanical ventilation, who were admitted to the ICU, who had a longer LOS, and who had a higher mRS at discharge (all *P* < 0.001). Furthermore, we found a positive correlation between NFL and time from admission to final blood draw (Spearman’s *r*: 0.50, *P* < 0.001); thus, we also performed similar analyses adjusting for time from admission to final blood draw (table S2) and observed results consistent with findings reported in Table 3.

**Table 3:** Associations between serum NFL and outcomes in patients with COVID-19. For descriptive summaries of NFL concentration and for ease of presentation, LOS was categorized using the sample median, whereas mRS at discharge was categorized using a predefined cutoff of interest. Associations of intubation, ICU admission, LOS, and mRS at discharge (all as independent variables) with NFL concentration (as the dependent variable) were evaluated using linear regression models (for the continuous mean NFL per patient, minimum NFL per patient, and maximum NFL per patient variables) and logistic regression models (for the binary occurrence of an NFL value >25th percentile and occurrence of an NFL value >75th percentile variables). Regression coefficients are interpreted as the change in the mean NFL outcome measure (on the natural logarithmic scale) corresponding to the presence of the given characteristic (intubation and ICU admission), to each doubling of LOS, and to each 1-unit increase in mRS at discharge. CI, confidence interval; CKD, chronic kidney disease. *P* values <0.0025 were considered as statistically significant after applying a Bonferroni correction for multiple testing.

**Association** **examined**	**Median (minimum, maximum) NFL** **or no. (%) for the given group**		**Association** **measure**	**Unadjusted analysis**		**Adjusting for age, sex, BMI,** **and CKD**	
				**Estimate (95% CI)**	***P* value**	**Estimate (95% CI)**	***P* value**
Association between intubation and	No intubation (*N* = 112)	Intubation (*N* = 30)					
Mean NFL per patient	19.7 (3.4, 972.7)	107.4 (15.9, 1538.4)	Regression coefficient	1.61 (1.16, 2.06)	<0.001	1.66 (1.24, 2.07)	<0.001
Minimum NFL per patient	16.6 (2.2, 585.6)	50.3 (3.1, 1538.4)	Regression coefficient	1.25 (0.78, 1.72)	<0.001	1.31 (0.87, 1.75)	<0.001
Maximum NFL per patient	22.2 (3.4, 1359.7)	111.5 (15.9, 2131.0)	Regression coefficient	1.75 (1.27, 2.24)	<0.001	1.79 (1.35, 2.24)	<0.001
NFL value >25th percentile	74 (66.1%)	30 (100.0%)	Odds ratio	N/A*	<0.001	N/A*	N/A*
NFL value >75th percentile	15 (13.4%)	19 (63.3%)	Odds ratio	11.17 (4.45, 28.03)	<0.001	10.64 (3.80, 29.80)	<0.001
							
Association between ICU admission and	No ICU admission (*N* = 88)	ICU admission (*N* = 54)					
Mean NFL per patient	18.7 (3.4, 972.7)	52.2 (4.8, 1538.4)	Regression coefficient	1.10 (0.69, 1.50)	<0.001	1.13 (0.76, 1.49)	<0.001
Minimum NFL per patient	15.8 (2.2, 585.6)	33.2 (3.1, 1538.4)	Regression coefficient	0.82 (0.41, 1.23)	<0.001	0.86 (0.48, 1.24)	<0.001
Maximum NFL per patient	20.4 (3.4, 1359.7)	64.5 (7.9, 2131.0)	Regression coefficient	1.23 (0.80, 1.66)	<0.001	1.26 (0.87, 1.65)	<0.001
NFL value >25th percentile*	55 (62.5%)	49 (90.7%)	Odds ratio	5.88 (2.13, 16.25)	<0.001	9.84 (3.00, 32.29)	<0.001
NFL value >75th percentile	10 (11.4%)	24 (44.4%)	Odds ratio	6.24 (2.67, 14.59)	<0.001	8.00 (3.08, 20.75)	<0.001
							
Association between LOS and	LOS ≤ 9 days (*N* = 79)	LOS > 9 days (*N* = 63)					
Mean NFL per patient	15.9 (3.4, 972.7)	47.8 (4.8, 1538.4)	Regression coefficient	0.81 (0.60, 1.01)	<0.001	0.74 (0.55, 0.93)	<0.001
Minimum NFL per patient	13.9 (2.2, 585.6)	33.4 (3.1, 1538.4)	Regression coefficient	0.66 (0.44, 0.87)	<0.001	0.60 (0.40, 0.80)	<0.001
Maximum NFL per patient	18.6 (3.4, 1359.7)	66.4 (7.9, 2131.0)	Regression coefficient	0.89 (0.67, 1.10)	<0.001	0.82 (0.62, 1.02)	<0.001
NFL value >25th percentile*	45 (57.0%)	59 (93.7%)	Odds ratio	5.34 (2.53, 11.24)	<0.001	4.90 (2.27, 10.62)	<0.001
NFL value >75th percentile	8 (10.1%)	26 (41.3%)	Odds ratio	3.17 (1.91, 5.24)	<0.001	3.24 (1.92, 5.47)	<0.001
							
Association between mRS at discharge and	mRS at discharge ≤3 (*N* = 109)	mRS at discharge >3 (*N* = 33)					
Mean NFL per patient	19.5 (3.4, 405.3)	91.9 (17.9, 1538.4)	Regression coefficient	0.56 (0.46, 0.67)	<0.001	0.54 (0.42, 0.66)	<0.001
Minimum NFL per patient	14.7 (2.2, 304.9)	47.8 (3.1, 1538.4)	Regression coefficient	0.49 (0.38, 0.61)	<0.001	0.45 (0.33, 0.58)	<0.001
Maximum NFL per patient	21.4 (3.4, 779.0)	105.0 (18.1, 2131.0)	Regression coefficient	0.60 (0.48, 0.71)	<0.001	0.58 (0.46, 0.71)	<0.001
NFL value >25th percentile*	71 (65.1%)	33 (100.0%)	Odds ratio	3.67 (2.17, 6.22)	<0.001	3.00 (1.71, 5.29)	<0.001
NFL value >75th percentile	16 (14.7%)	18 (54.5%)	Odds ratio	2.27 (1.66, 3.11)	<0.001	3.04 (2.02, 4.58)	<0.001

*When evaluating the association between intubation and NFL value >25th percentile, logistic regression was not possible owing to the occurrence of a zero cell count. Therefore, the *P* value in unadjusted analysis results from Fisher’s exact test, and multivariable analysis was not performed.

### Longitudinal serum NFL concentrations in hospitalized patients with COVID-19 and their association with treatment type

Among the 142 patients with COVID-19, longitudinal NFL data were available for 100 individuals for whom serum NFL was measured for up to 17 time points. As anticipated, temporal changes in NFL differed among patients, and this was true of the 35 patients with longitudinal NFL measures and for whom mean NFL concentrations were considered elevated (Fig. 1C). For instance, in some individuals, serum NFL rose over time, whereas for others NFL remained relatively consistent or fluctuated to varying degrees.

Since patients were administered a range of potential therapies including monoclonal antibody treatment, remdesivir, dexamethasone, and convalescent plasma therapy (Table 1), we next examined whether these treatments influenced NFL. Comparisons of serum NFL according to specific treatments for COVID-19 are displayed in Table 4. After adjusting for age, sex, BMI, and time from admission to blood draw, as well as correcting for multiple analyses (*P* < 0.0063 considered as significant), a tendency toward lower serum NFL in patients who received remdesivir was observed in multivariable analysis (final NFL concentration per patient, *P* = 0.008; Table 4).

**Table 4:** Comparison of serum NFL concentrations according to COVID-19 treatment. Regression coefficients, 95% CIs, and *P* values result from linear regression models, where NFL was the dependent variable. Regression coefficients are interpreted as the difference in mean NFL concentration (on the natural logarithmic scale) between patients who had the given treatment and patients who did not have the given treatment. *P* values <0.0063 were considered as statistically significant after applying a Bonferroni correction for multiple testing.

**COVID-19** **treatment**	***N***	**Median (minimum,** **maximum) NFL** **concentration**	**Adjusting for time from admission to** **blood draw**		**Adjusting for age, sex, BMI, and time from** **admission to blood draw**	
			**Regression** **coefficient (95% CI)**	***P* value**	**Regression** **coefficient (95% CI)**	***P* value**
Monoclonal antibody treatment						
First NFL value per patient						
No treatment	125	22.5 (2.9, 1538.4)	1.00 (reference)	N/A	1.00 (reference)	N/A
Treatment	17	20.0 (3.5, 647.3)	−0.34 (−0.91, 0.23)	0.29	−0.47 (−1.00, 0.05)	0.074
Final NFL value per patient						
No treatment	107	31.1 (2.2, 1538.4)	1.00 (reference)	N/A	1.00 (reference)	N/A
Treatment	35	33.4 (3.1, 1465.8)	−0.38 (−0.85, 0.09)	0.12	−0.39 (−0.84, 0.07)	0.094
Remdesivir treatment						
First NFL value per patient						
No treatment	92	21.2 (2.9, 872.3)	1.00 (reference)	N/A	1.00 (reference)	N/A
Treatment	50	23.7 (3.4, 1538.4)	−0.25 (−0.68, 0.18)	0.25	−0.24 (−0.64, 0.15)	0.23
Final NFL value per patient						
No treatment	42	32.3 (3.4, 1359.7)	1.00 (reference)	N/A	1.00 (reference)	N/A
Treatment	100	32.1 (2.2, 1538.4)	−0.47 (−0.89, −0.05)	0.029	−0.56 (−0.97, −0.15)	0.008
Dexamethasone treatment						
First NFL value per patient						
No treatment	103	21.4 (2.9, 1538.4)	1.00 (reference)	N/A	1.00 (reference)	N/A
Treatment	39	22.5 (3.4, 872.3)	0.27 (−0.16, 0.69)	0.22	0.26 (−0.13, 0.64)	0.19
Final NFL value per patient						
No treatment	66	29.3 (2.2, 1538.4)	1.00 (reference)	N/A	1.00 (reference)	N/A
Treatment	76	37.8 (3.1, 1465.8)	−0.11 (−0.51, 0.29)	0.59	−0.08 (−0.46, 0.31)	0.69
Convalescent plasma treatment						
First NFL value per patient						
No treatment	119	20.3 (2.9, 1538.4)	1.00 (reference)	N/A	1.00 (reference)	N/A
Treatment	23	33.4 (9.4, 647.3)	−0.24 (−0.82, 0.35)	0.43	−0.25 (−0.78, 0.28)	0.35
Final NFL value per patient						
No treatment	107	27.1 (2.2, 1538.4)	1.00 (reference)	N/A	1.00 (reference)	N/A
Treatment	35	40.0 (3.1, 1186.2)	−0.01 (−0.46, 0.45)	0.98	−0.02 (−0.41, 0.45)	0.93

## DISCUSSION

COVID-19 is associated with diverse neurological injuries, but questions regarding their incidence, severity, and long-term consequences, and whether such injuries can be mitigated by acute intervention, remain unanswered (*21*, *22*). Toward addressing these important questions, we examined the utility of serum NFL in determining the frequency, severity, and clinical consequences of neuronal injury in hospitalized patients with COVID-19. We show that serum NFL is elevated in patients with COVID-19 and that ~34% of patients have mean NFL concentrations of at least 3 SDs above the group mean of control individuals. In addition, elevated serum NFL correlates with worse clinical outcomes, such as the need for mechanical ventilation (intubation), ICU admission, longer lengths of hospitalization, and worse functional outcomes. We further observed a tendency of lower serum NFL in patients with COVID-19 treated with remdesivir. In aggregate, these findings suggest that a considerable proportion of hospitalized patients with COVID-19 suffer neuronal injury, the degree of which associates with disease severity.

We reported that patients with COVID-19 can develop a myriad of neurologic symptoms including headaches, encephalopathy, and seizures (*13*), providing indication for head imaging. Nevertheless, the great majority of patients with COVID-19 (86.7%) show no intracranial abnormalities by imaging (*13*). It also bears mentioning that retrospective studies using brain imaging approaches in patients with COVID-19 suffer from selection bias, in particular omitting patients with mild or no obvious neurological symptoms and patients too unstable or having contraindications to undergo magnetic resonance imaging. This selection bias, combined with the uncertainty of the sensitivity of brain imaging to neuroaxonal injury (*12*), suggests that neuronal injury or neurodegeneration in patients with COVID-19 may be underappreciated based on imaging studies alone. Given findings from the present study, we believe that measuring serum NFL in patients with COVID-19 will facilitate the detection of neuronal injury that may otherwise be overlooked.

Our findings that serum NFL is elevated in our cohort of 142 patients hospitalized with COVID-19, and that higher serum NFL associates with worse clinical outcomes, are in line with previous reports. For example, one study observed elevated serum NFL in 28 patients with mild-to-moderate COVID-19 (*23*), whereas another found that, among 47 patients with COVID-19, plasma NFL was higher in the 18 patients with severe disease (*24*). Yet, another reported serum NFL to be elevated in 29 critically ill patients with COVID-19 and to positively associate with an unfavorable outcome (*25*). Last, cerebrospinal fluid (CSF) NFL was elevated in two of six hospitalized patients with moderate or severe COVID-19 (*26*). Our data are also congruent with the growing consensus that SARS-CoV-2 causes potentially damaging neurological problems. However, how this occurs remains unclear. Similarly to other respiratory viruses that have neuroinvasive capacities (*27*, *28*), SARS-CoV-2 can spread to the central nervous system (*17*, *18*). Postulated pathways by which this occurs include its retrograde axonal transport along the sensory and olfactory nerves in the cribriform plate, its invasion of endothelial cells by interacting with the angiotensin-converting enzyme 2 receptor, and its ability to alter tight junction proteins formed by endothelial cells (*29*). Nevertheless, despite the ability of SARS-CoV-2 to invade the brain, the neurological signs and symptoms it causes are believed to more likely result from systemic reactions such as hypoxemia, hypercoagulability, systemic inflammation, and multiorgan failure (*18*, *26*, *30*).

Therapies available for COVID-19 include dexamethasone (*31*), monoclonal antibody toward the spike protein of SARS-CoV-2 (*32*), remdesivir (a viral replication inhibitor) (*33*, *34*), and convalescent plasma (*35*, *36*). In our cohort of patients with COVID-19, a tendency of lower serum NFL concentrations was seen with the use of remdesivir compared to its non-use. Although this association needs to be confirmed in larger patient cohorts and independently replicated, these findings underscore the utility of incorporating subacute measurements of NFL during hospitalization and in randomized drug trials.

Strengths of our study include the relatively substantial number of patients with COVID-19, the longitudinal examination of serum NFL, and our analysis of associations of NFL with clinical outcomes and treatment type. Our study also has limitations. Most patients in our cohort were hospitalized for a considerable length of time such that data for patients with shorter hospital stays (due to less severe disease) were comparatively limited. There were also delays from time of suspected infection or the time of first symptoms to blood draw. Our analyses used the time of hospital admission as the baseline time point; however, the length of time from symptom onset to admission likely varies among patients. Last, our analyses assessing associations of COVID-19 treatments with serum NFL should be viewed as exploratory. The small sample sizes together with differences in timing of treatment in relation to symptom onset, in severity of symptoms once treatment was initiated, and in duration of treatment from its initiation to blood draw for NFL measurements may have hampered our efforts to detect associations of interest. To rigorously address this question would require a randomized trial in which NFL concentrations are longitudinally measured in patients before and after treatment.

Overall, we show that serum NFL was elevated in hospitalized patients with COVID-19 and correlated with worse clinical outcomes. These data further cement the field’s recognition of the neurological manifestations caused by SARS-CoV-2 infection. Nevertheless, our understanding of the long-term implications is limited. It has been shown that patients previously hospitalized with COVID-19 display a wide array of neurological symptoms months after discharge (*10*, *12*, *37*), but few studies have systematically followed patients over time, likely because of the inherent difficulties in doing so. However, on the basis of our present data, we posit that longitudinal measurements of serum NFL would provide an efficient means to identify and quantify neurological injury in hospitalized patients with COVID-19. Serum NFL may also be useful for monitoring end-stage organ disease progression and recovery, aiding in the identification of risk factors and clinical features that contribute to COVID-19–associated neurological signs, and indicating neuroaxonal injury in COVID-19 drug trials.

## MATERIALS AND METHODS

### Study design

The goal of this study was to investigate serum NFL as a biomarker of neuroaxonal injury in patients with COVID-19. NFL was measured using the NF-Light digital immunoassay from Quanterix. Our primary analyses were to determine whether serum NFL is elevated in hospitalized patients with COVID-19 and whether NFL associates with clinical outcomes and treatment type. We included 142 patients with COVID-19 (no randomization) for whom serum (488 samples) was collected cross-sectionally or longitudinally during hospitalization. Our study also included serum from 55 healthy controls. NFL was measured in a blinded manner. Sample sizes were based on what was available when the study was initiated and not on sample size calculations. Biological samples were obtained when residual blood was available from patients with approval by the ethics committee.

### Study subjects

A total of 488 serum samples from 142 patients hospitalized with COVID-19 were included in this study. The Mayo Clinic Neurological, Vascular and Neurovascular Events With SARS-CoV-2 Study [MC NEWS; Institutional Review Board (IRB) #20-003457] was queried to identify a cohort of individuals with COVID-19. MC NEWS included patients across the three major campuses of Mayo Clinic and the Mayo Clinic Health System, with hospitals in Arizona, Florida, Minnesota, and Wisconsin. Fifty-five healthy controls without COVID-19 and no neurological condition were additionally included. This group was composed of 22 healthy individuals from the general population, 19 healthy spouses or caregivers of patients with ataxia (*N* = 15) or amyotrophic lateral sclerosis (ALS; *N* = 4), and 14 unaffected relatives of patients with ataxia (*N* = 4) or ALS (*N* = 10) and who lacked disease-associated gene mutations. Serum samples were obtained under IRB approval through the following protocols: “Investigating biomarkers, disease mechanisms and treatments for spinocerebellar ataxia and nucleotide repeat diseases,” IRB#17-006033; “Investigating the Genetic and Phenotypic Presentation of Spinocerebellar Ataxia and Nucleotide Repeat Diseases,” IRB#16-009414; “Biospecimen Biorepository for the Study of ALS, ALS-FTD and Similar Neurodegenerative Disorders,” IRB#13-004314; “Pilot Evaluation of Neurofilament Heavy Form (NF-H) as a Potential Biomarker of Axonal Loss in Amyotrophic Lateral Sclerosis (ALS),” IRB#10-003592; “Clinical & Genetic Studies in ALS, Suspected ALS, and Other Neurodegenerative Motor Neuron Disorders,” IRB#07-005711; “Biospecimen Collection to Investigate the Causes of ALS,” IRB#15-001187; “The DIALS (Dominant Inherited ALS) Network,” IRB#2017P000485; and “COVID-19 cytokine storm project,” IRB#20-003661. For patients with COVID-19, the following demographic and clinical information was abstracted using the shared electronic medical record (Epic): age, sex, BMI, CKD status, and COVID-19 treatment status for monoclonal antibody therapy, remdesivir, dexamethasone, and convalescent plasma. The following patient outcomes were collected: need for mechanical ventilation (intubation), ICU admission, LOS, mRS at discharge, vital status on follow-up, and death. The mRS is a seven-level ordered categorical scale commonly used in stroke studies that ranges from 0 (fully independent; no symptom) to 6 (death) (*38*). Death (not restricted to hospitalization) was summarized descriptively but was not analyzed as an outcome in association analyses owing to the small number of patients who died (13 of 142) and because death is included in the mRS at discharge outcome (note: 2 of the 13 patients who died did so after discharge; hence, only 11 patients had an mRS score of 6 at discharge). Age, race, and sex were collected for the control individuals.

### NFL measurements

Serum NFL concentrations were measured in duplicate in a blinded fashion on a Simoa HD-X analyzer using an NF-Light digital immunoassay (Quanterix, catalog no. 103186) according to the manufacturer’s instructions. Briefly, samples were thawed and cleared by centrifugation at 10,000*g* for 5 min, transferred to 96-well plates, and run in duplicate using a 4× instrument dilution. Four samples were included in each run to monitor potential variability among assays, along with appropriate calibrators and controls provided by the manufacturer. NFL concentrations were interpolated from the calibration curve using a 1/y2 weighted four-parameter logistic curve fit. Samples with NFL concentrations that exceeded the range of the assay were retested using an appropriate at-bench dilution in addition to the 4× instrument dilution. Samples with coefficients of variation above 20% were also retested.

For all 55 control subjects, NFL concentrations were measured in serum collected at a single time point. For patients with COVID-19, NFL was measured in serum collected at a single time point for 42 individuals (29.6% of patients) and in serum collected at multiple time points for the remaining patients. For patients who underwent longitudinal serum collection, the median number of NFL measurements was 4 (range, 2 to 17).

### Statistical analysis

Continuous variables were summarized with the sample median and range. Categorical variables were summarized with number and percentage of patients. Comparisons of NFL concentrations between controls and patients with COVID-19 were made using a Wilcoxon rank sum test in unadjusted analysis and using a stratified van Elteren Wilcoxon rank sum test (*39*) in adjusted analysis, where the test was stratified by both age as a four-level categorical variable (based on sample quartiles) and sex. For comparisons of NFL between the control group and patients with COVID-19, single–time point NFL concentrations of controls were compared to the following five NFL values in patients: (i) NFL in serum from the first blood draw, (ii) NFL in serum from the last blood draw, (iii) mean NFL concentration in all serum samples for a given patient, (iv) minimum NFL concentration per patient, and (v) maximum NFL concentration per patient. In patients alone, we also assessed the strength of association between the length of time from admission to blood draw and NFL concentrations by estimating Spearman’s correlation coefficient and by obtaining a *P* value from a mixed effects linear regression model that included a random effect for patients. NFL was examined on the natural logarithmic scale in this and all subsequently described analyses owing to its skewed distribution.

When assessing associations of NFL concentrations with outcomes (need for mechanical ventilation/intubation, ICU admission, LOS, and mRS at discharge), we considered the five following patient-specific NFL variables: (i) mean NFL concentration per patient, (ii) minimum NFL concentration per patient, (iii) maximum NFL concentration per patient, (iv) occurrence of an NFL concentration >25th percentile, and (v) occurrence of an NFL concentration >75th percentile. The 25th and 75th percentile cutoffs were calculated using all NFL concentrations in our cohort of patients with COVID-19.

Given that outcomes often occurred either before measurement of any NFL values (need for mechanical ventilation/intubation or ICU admission) or at a very similar time point as measurement of some NFL values (LOS and mRS at discharge), performing an analysis that examined the ability of NFL to predict these outcome measures was not possible given the data. Therefore, to assess whether NFL associates with poor outcomes in patients with COVID-19, we assessed associations between outcomes (intubation, ICU admission, LOS, and mRS at discharge) and each of the five aforementioned NFL variables using linear regression models (mean, minimum, and maximum NFL per patient) and logistic regression models (NFL >25th percentile and NFL >75th percentile), where the NFL variables were examined as the dependent variables in the regression models and NFL values were included regardless of temporal relationship to time of start of mechanical ventilation (intubation) or time of ICU admission. Regression coefficients and 95% confidence intervals (CIs) were estimated from linear regression models, whereas odds ratios (ORs) and 95% CIs were estimated from logistic regression models. Unadjusted models were first examined, and these were followed by multivariable models that were adjusted for age, sex, BMI, and CKD. The latter was included given that blood NFL concentrations are affected by renal function (*40*). Length of time from admission to blood draw was not initially taken into account in multivariable models because this variable can, to some extent, also be thought of as an outcome measure (because a longer time from admission to blood draw indicates a patient with a longer hospitalization and likely worse outcomes) and therefore may be on the causal pathway between NFL and outcomes (*41*). However, in a secondary analysis, we did examine the sensitivity of our results to additional multivariable adjustment for length of time from admission to final blood draw.

We examined whether specific COVID-19 treatments (monoclonal antibody, remdesivir, dexamethasone, and convalescent plasma) associated with a lower or higher serum NFL as follows. First, to satisfy the statistical assumption of independent measurements, we performed two separate analyses using a single NFL value per patient (because many patients had multiple NFL values), with the first analysis using the NFL concentration from the first blood draw for each patient and the second analysis using the NFL concentration in the final blood draw for each patient. For each of these two analyses, we compared serum NFL concentrations between patients who did and did not receive the given treatment (only NFL values that were measured after the given treatment were used for patients in a given “treatment” group) using linear regression models. Owing to the very strong confounding influence of time from admission to blood draw, we first adjusted models for this variable alone and then subsequently additionally adjusted for age, sex, and BMI. Regression coefficients and 95% CIs were estimated.

We used a Bonferroni correction for multiple testing separately for each family of similar statistical tests. After applying this correction, *P* values <0.0025 were considered as statistically significant when evaluating associations of NFL with outcomes (four different outcomes were examined for five NFL variables), and *P* values <0.0063 were considered as statistically significant when assessing associations of NFL with COVID-19 treatment (four different treatments were examined for two NFL variables). *P* values <0.05 were considered as statistically significant in all other analyses. All statistical tests were two-sided and were performed using SAS (version 9.4; SAS Institute Inc.).

## SUPPLEMENTARY MATERIALS

stm.sciencemag.org/cgi/content/full/scitranslmed.abi7643/DC1
Tables S1 and S2

## Appendix

View/request a protocol for this paper from *Bio-protocol*.
